# Myocardial function in COVID-19 patients after hospital discharge: a descriptive study comparing the first and second ‘wave’ patients

**DOI:** 10.1007/s10554-022-02590-3

**Published:** 2022-04-16

**Authors:** T. W. Elffers, M. A. de Graaf, M. V. Regeer, S. Omara, M. J. Schalij, G. H. Groeneveld, A. H. E. Roukens, J. J. M. Geelhoed, M. L. Antoni

**Affiliations:** 1 Department of Cardiology, Leiden University Medical Centre, Albinusdreef 2, 2333ZA Leiden, The Netherlands; 2 Department of Infectious Diseases and Internal Medicine, Leiden University Medical Centre, Leiden, The Netherlands; 3 Department of Pulmonology, Leiden University Medical Centre, Leiden, The Netherlands

**Keywords:** COVID-19, Out-patient clinic, Echocardiography, Strain

## Abstract

In hospitalized COVID-19 patients, myocardial injury and echocardiographic abnormalities have been described. The present study investigates cardiac function in COVID-19 patients 6 weeks post-discharge and evaluates its relation to New York Heart Association (NYHA) class. Furthermore cardiac function post-discharge between the first and second wave COVID-19 patients was compared. We evaluated 146 patients at the outpatient clinic of the Leiden University Medical Centre. NYHA class of II or higher was reported by 53% of patients. Transthoracic echocardiography was used to assess cardiac function. Overall, in 27% of patients reduced left ventricular (LV) ejection fraction was observed and in 29% of patients LV global longitudinal strain was impaired (> − 16%). However no differences were observed in these parameters reflecting LV function between the first and second wave patients. Right ventricular (RV) dysfunction as assessed by tricuspid annular systolic planar excursion (< 17 mm) was present in 14% of patients, this was also not different between the first and second wave patients (15% vs. 12%; p = 0.63); similar results were found for RV fraction area change and RV strain. Reduced LV and RV function were not associated with NYHA class. In COVID-19 patients at 6 weeks post-discharge, mild abnormalities in cardiac function were found. However these were not related to NYHA class and there was no difference in cardiac function between the first and second wave patients. Long term symptoms post-COVID might therefore not be explained by mildly abnormal cardiac function.

**Supplementary Information:**

The online version contains supplementary material available at 10.1007/s10554-022-02590-3.

## Introduction

The first Coronavirus disease 2019 (COVID-19) case was reported in December 2019 and since then there have been several ‘waves’ in this pandemic. As in other respiratory tract infections, COVID-19 has been associated with myocardial damage evaluated by elevated troponin T levels and cardiac dysfunction on echocardiography [1–3]. Several mechanisms contribute to the myocardial damage caused by COVID-19, among which direct viral damage to the myocardium, cytokine-mediated damage, and damage caused by the hypercoagulable state associated with COVID-19 [4]. Furthermore, an increased afterload associated with pulmonary disease may cause RV dysfunction. Mainly in the hospitalized population, echocardiographic abnormalities are reported, e.g., right ventricular (RV) dilation and impaired function, whereas left ventricular (LV) function is often preserved [5–8]. Importantly, echocardiographic abnormalities during hospitalization are associated with higher mortality [9–12]. Currently, there is still limited information available regarding the mid- and long-term cardiac consequences after discharge of COVID-19 patients. Some studies report no important long-term cardiac abnormalities and other studies do observe clinical or subclinical cardiac dysfunction during follow-up [13–16]. There is an important subgroup of post COVID-19 patients that experiences long term symptoms, e.g., tiredness or shortness of breath. However, whether these symptoms are related to cardiac dysfunction post-discharge is unclear.

The COVID-19 pandemic is often described in several ‘waves’. The first wave was from March to June 2020 and the second from July to December 2020. Since the start of the pandemic, rapid improvement in early diagnosis, treatment and management strategies have been observed resulting in lower mortality rates. Therefore, in the second wave, due to increased awareness and earlier presentation to the hospital, admitted patients were less severely affected in comparison to the first wave. Several studies have compared patient characteristics and clinical outcomes between the first and second wave, however few studies have compared cardiac sequelae of COVID-19 infection between these two waves [17, 18].

Therefore, the aim of the current study was to evaluate the cardiac function in patients after hospitalization with COVID-19 in relation to persisting symptoms. Furthermore, clinical and echocardiographic parameters were compared between the first and second wave COVID-19 patients.

## Methods

### Design and study population

Patients admitted with PCR confirmed SARS-CoV-2 infection at the Leiden University Medical Centre in 2020 were considered eligible for the present study. In a previous study, the short-term outpatient follow-up of these patients was described [19]. As part of regular follow-up, patients were planned for an outpatient clinic visit 6 weeks after discharge to assess residual complaints and potential cardiac sequelae after serious COVID-19 infection. Patients were divided into two groups: the first wave refers to patients hospitalized during March–June 2020 and the second wave refers to patients hospitalized during July–December 2020 [20]. Furthermore, an analysis was made according to COVID-19 disease severity. Severe disease was defined as patients in need of Intensive care unit (ICU) support. Patients with a known cardiac history were excluded from the present analysis.

The New York Heart Association (NYHA) classification was used to evaluate residual complaints of dyspnea. Clinical characteristics were collected from the departmental cardiology information system (EPD-vision; Leiden University Medical Centre (LUMC), Leiden, the Netherlands). The hospital’s ethical review board approved the study. All patients admitted to the hospital were given a letter which stated that their data could be used for research purposes, and that they could opt out if they disagreed. None of the admitted patients have declined consent.

### Transthoracic echocardiography

Patients underwent transthoracic echocardiography (TTE) 6 weeks after hospital admission. TTE was performed in the left lateral decubitus position at rest using commercially available ultrasound systems (Vivid E95, General Electronics Healthcare, Horten, Norway). Parasternal, apical, subcostal and suprasternal views were obtained. Two-dimensional and Doppler data were digitally stored and retrospectively evaluated using EchoPac (General Electronics Healthcare, Horten, Norway).

Chamber quantification and systolic function was assessed according to current recommendations [21]. LV end-diastolic and end-systolic volumes were measured in apical four- and two-chamber views and LV ejection fraction (LVEF) was calculated using Simpsons biplane method. In addition, speckle tracking derived global longitudinal strain (GLS) was measured from apical three-, four- and two-chamber views. RV end-diastolic diameter was measured in the apical four-chamber view. RV function was assessed by measuring tricuspid annular systolic planar excursion (TAPSE) and tricuspid lateral annular systolic velocity wave derived from tissue doppler imaging (Sʹ). In addition, RV fractional area change (RV FAC) and RV free wall strain were measured on the apical RV-focused four-chamber view. Diastolic function was graded on a semiquantitative scale (grade 0–3) using an integrated approach measuring E/A ratio, Eʹ, E/Eʹ, left atrial volume index (LAVi) and tricuspid regurgitation gradient following current guidelines. LVEF was divided in four categories (> 52% for males or > 54% for females; 40–52/54%; 30–40% and < 30%). LV GLS was divided in normal and abnormal (≤ − 16% resp. >  − 16%), as was TAPSE (> 17 mm resp. ≤ 17 mm), RV FAC (> 35% resp. ≤ 35%) and RV free wall strain (≤ -23% resp. > − 23%) and Sʹ (> 10 cm/s (cm/s) resp. ≤ 10 cm/sec) [22–24].

### Statistical analysis

Statistical analysis were performed using IBM SPSS version 25.0. Continuous variables were reported as mean ± standard deviation or as median (interquartile range). Categorical variables were reported as percentages. Continuous and categorical variables were compared using Students t-test and Chi square test, respectively.

## Results

Characteristics of the study population are presented in Table 1. Mean age of the patients was 61.1 (SD 12.2) years, 64% were male, and mean BMI was 28.6 ± 5.5 kg/m^2^. COVID-19 patients in the first wave showed higher maximum CRP (190.8 ± 133.3 vs. 99.1 ± 97.8 mg/L; p < 0.01), higher percentage of patients with pulmonary embolism (20 vs. 8%; p = 0.03)) in comparison to patients in the second wave. In the total cohort, 35 (24%) patients were admitted to the ICU, of these patients 29 (39.7%) were admitted during the first wave and 6 (8,2%) during the second wave (P < 0.01). Patients with admitted to the ICU had a higher CRP (296 vs 97%, P < 0.001) and more often presented with pulmonary embolism (34 vs 8%, p < 0.001) (see Supplementary Table 1 for the baseline characteristics stratified according to disease severity).

**Table 1:** Baseline characteristics of the study population

	All (n = 146)	First wave (n = 73)	Second wave (n = 73)	p-value
Age, years	61.1 (12.2)	59.2 (12.3)	63.0 (11.9)	0.06
Sex, men, %	63.7	63.0	64.4	0.86
BMI, kg/m^2^	28.6 (5.5)	28.1 (4.7)	29.2 (6.1)	0.21
*History of*				
Hypertension, %	34.9	32.9	37.0	0.60
Diabetes, %	23.3	23.3	23.3	1.00
Cardiovascular disease, %				
Atrial fibrillation/ atrial flutter	6.8	5.5	8.2	0.51
CVA/TIA	4.8	8.2	1.4	0.05
PVD	2.7	4.1	1.4	0.31
CKD, %	8.2	8.2	8.2	1.00
Smoking, %	15.1	11.0	19.2	0.17
*In hospital*				
CRP maximum, mg/L	145.3 (125.4)	190.8 (133.3)	99.1 (97.8)	** < 0.01**
Pulmonary embolism, %	14.4	20.5	8.2	**0.03**
Intensive care admission, %	24	39.7	8.2	** < 0.01**
Troponin T max ng/L	15.8 (16.3)	17.0 (17.5)	13.7 (13.7)	0.30

Data are presented as mean (SD) or percentage

*BMI* body mass index, *CKD* chronic kidney disease, *CRP* C-reactive protein, *CVA* cerebrovascular accident, *PVD* peripheral vascular disease, *TIA* transient ischemic attack

There was no difference in mean maximum troponin T level during admission (17.0 vs. 13.7 ng/L; p = 0.30) between the first and second wave group.

**Fig. 1:**
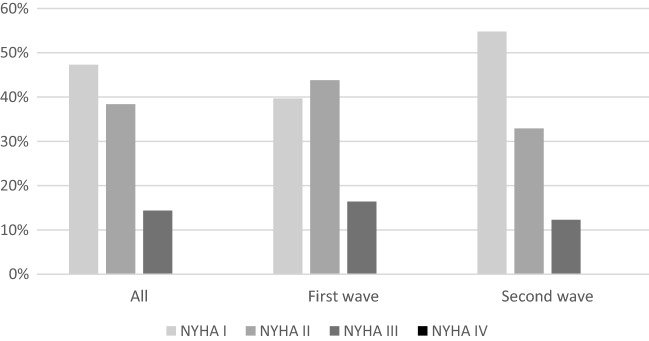
NYHA class at visit 6 weeks post-discharge. *NYHA* New York Heart Association

During follow-up at the out-patient clinic 6 weeks post discharge, 47% of patients were NYHA class I, 38% NYHA class II, 14% of patient NYHA class III and 0% NYHA class IV; as shown in Fig. 1**.** In the second wave patients, a higher percentage had a better functional status as reflected by NYHA class (e.g., 40% NYHA class I in first wave vs. 55% NYHA class I in second wave), however these differences were not statistically significant (p = 0.11).

Table 2 shows echocardiographic parameters at 6 weeks follow-up after hospitalization with COVID-19.

**Table 2:** Echocardiography at 6 weeks post discharge

	*All*	*First wave (n* = *73)*	*Second wave (n* = *73)*	p-value
LVESV, mL	41.0 (18.8)	42.7 (15.8)	39.3 (21.5)	0.29
LVEDV, mL	91.7 (33.1)	97.0 (30.3)	86.3 (35.0)	0.05
LVEF, %	56.2 (6.7)	56.6 (5.1)	55.8 (8.0)	0.47
> 52(M), > 54(F)	72.6	76.7	68.5	
40–52(M)/54(F)	24.7	23.3	26.0	
30–40	0.7	0	1.4	
< 30	0.7	0	1.4	
LV GLS, %	− 17.0 (2.5)	− 16.5 (2.3)	− 17.5 (2.6)	0.03
				0.16
≤ − 16	70.5	65.3	76.1	
> − 16	29.5	34.7	23.9	
RVEDD, mm	34.4 (4.8)	34.0 (4.5)	34.8 (5.1)	0.32
TAPSE, mm	21.0 (3.3)	21.0 (2.9)	21.0 (3.6)	0.90
				0.63
≤ 17	13.7	15.1	12.3	
> 17	86.3	84.9	87.7	
Sʹ, cm/sec	13.5 (3.0)	13.0 (3.0)	13.9 (3.0)	0.08
				0.97
≥ 10	15.8	15.7	15.9	
< 10	84.1	84.2	84.0	
RV FAC, %	42.9 (8.6)	42.2 (8.0)	43.6 (9.3)	0.35
				0.39
≤ 35	18.1	20.8	15.3	
> 35	81.9	79.2	84.7	
RV strain, %	− 23.6 (4.9)	− 24.1 (4.3)	− 23.1 (5.5)	0.22
				0.33
≤ − 23	56.9	60.9	52.5	
> − 23	43.1	39.1	47.5	
Diastolic dysfunction grade, %				0.99
0	59.6	58.9	60.3	
I	37.7	38.4	37.0	
II	2.7	2.7	2.7	
III	0	0	0	
E/A	1.0 (0.5)	1.0 (0.5)	0.9 (0.4)	0.16
Eʹ average, cm/s	8.9 (3.1)	9.0 (3.1)	8.9 (3.1)	0.81
E/Eʹ	7.6 (3.2)	7.4 (3.4)	7.8 (3.0)	0.42
LAVI, ml/m^2^	28.7 (10.3)	28.4 (9.6)	29.0 (11.1)	0.69
TR gradient				0.38
≤ 34 mmHg	94.7	96.4	92.3	
> 34 mmHg	5.3	3.6	7.7	

Data are presented as mean (SD), or as percentage

*LAVI* left atrial volume index, *LVEDV* left ventricular end-diastolic volume, *LVESV* left ventricular end-systolic volume, *LVEF* left ventricular ejection fraction, *LV GLS* left ventricular global longitudinal strain, *RVEDD* right ventricular end-diastolic diameter, *RV FAC* right ventricular fraction area change, *RV strain* right ventricular strain, *TAPSE* tricuspid annular plane systolic excursion, *TR gradient* tricuspid regurgitation gradient

### Left ventricular function

**Fig. 2:**
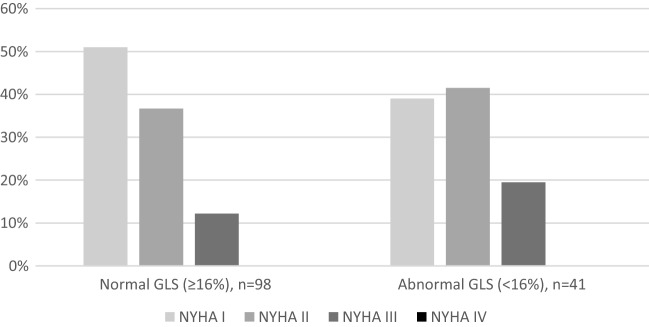
NYHA class at visit 6 weeks in patients with normal or abnormal GLS. *GLS* global longitudinal strain, *NYHA* New York Heart Association

**Fig. 3:**
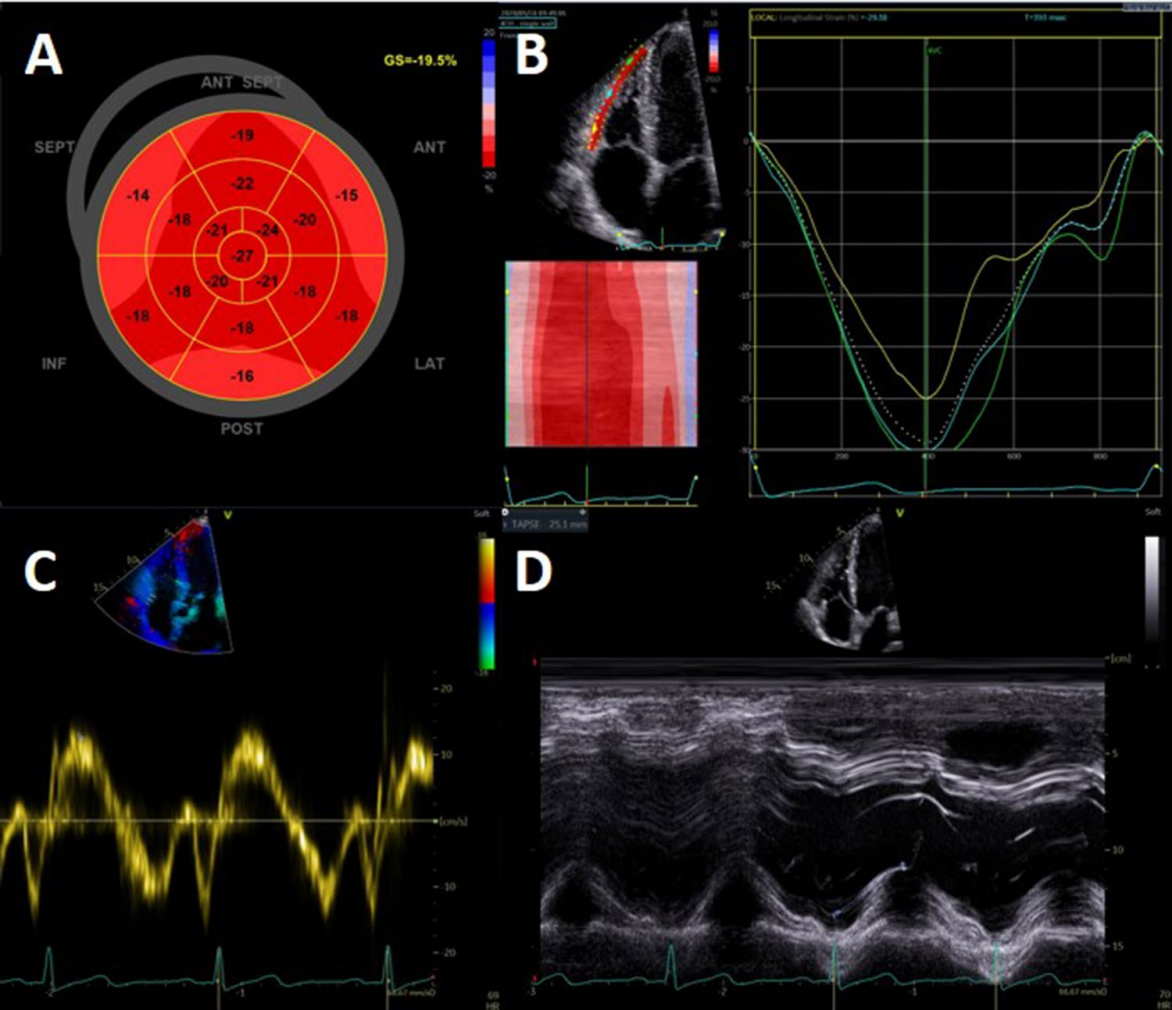
Case example of a COVID-19 patient with normal myocardial function. Case example of a 44-year old patient without prior medical history who had been admitted to the ICU for COVID-19 in the first wave and was on mechanical ventilation for 5 days. At the out-patient clinical he has persisting dyspnoea symptoms (NYHA II). Echocardiography shows no significant abnormalities. LVEF was 58% and LV GLS was normal (− 19%). Right ventricular function is preserved (TAPSE 25 mm,Sʹ 12 cm/sec, RV strain − 29)

**Fig. 4:**
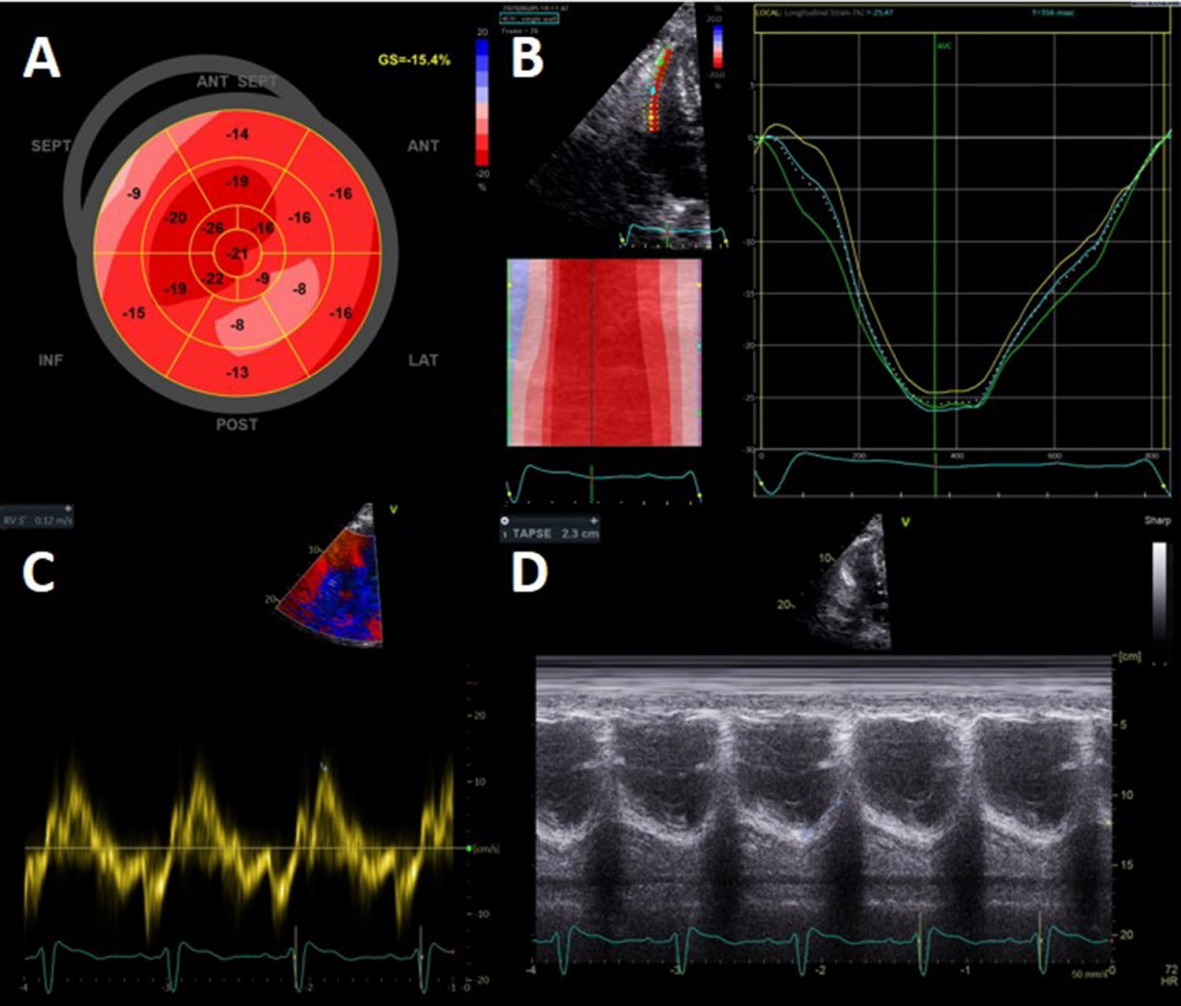
Case example of a COVID-19 patients with mild echocardiographic abnormalities. Example of a 75 year old patient with a history of diabetes and prostate cancer, he was admitted to the general ward for COVID-19 for 6 days in the first wave. During admission there were signs of cardiac injury (i.e., elevated troponin). At the out-patient clinical the patient has residual NYHA II dyspnoea symptoms. Echocardiography showed a mildly reduced left ventricular function (LVEF 54%, GLS − 15%). RV function was normal (TAPSE 23 mm, RV Sʹ 12 cm/sec, RV strain − 25%)

In the total study population, 73% of patients had a normal LVEF, defined as > 52% in men or > 54% in women. 25% of patients had a LVEF of 40–52/54% and only 1 patient had a LVEF between 30 and 40% and in 1 patient LVEF was < 30%. There were no statistically significant differences between the first and second wave regarding LVEF. In 30% of patients, LV-GLS was reduced (> 16%). Regarding LV GLS, there was also no significant difference between the first and second wave (abnormal LV GLS 35% vs. 24%; p = 0.16). An abnormal LV GLS was not associated with persisting symptoms assessed by NYHA class at 6 weeks follow-up (p = 0.35), as shown in Fig. 2. Grade I diastolic dysfunction was present in 38% of all patients, grade II in 3% of patients (0% grade III). Figures 3 and 4 demonstrate two cases of patients with persisting symptoms despite only mild abnormalities were obsvered on echocardiography.

### Right ventricular function

In the total study population, 14% of patients had RV dysfunction as assessed by TAPSE less than 17 mm, 15% in the first wave vs. 12% in the second wave (p = 0.63). RV FAC ≤ 35% was present in 18% of all patients; 21% in the first wave vs. 15% in the second wave (p = 0.39). Mean RV free wall strain was -23.6% in all patients; -24.1% in the first wave vs. -23.1% in the second wave (p = 0.22). In 5% of all patients tricuspid regurgitation gradient was > 34 mmHg; 4% in the first wave vs. 8% in the second wave (p = 0.38).

The echocardiography parameters were stratified according to disease severity (Table 3). No differences in echo cardiography at 6 weeks could be demonstrated between patients admitted to the ICU or the general ward.

**Table 3:** Echocardiography at 6 weeks post discharge according to disease severity

	ICU patients (n = 35)	Non-ICU patients (n = 111)	P-value
LVESV, mL	42.2 (17.2)	40.6 (19.4)	0.674
LVEDV, mL	95.8 (32.2)	90.4 (33.4)	0.412
LVEF, %	56.3 (5.1)	56.2 (7.1)	0.977
			0.861
> 52(M), > 54(F)	74.3	72.1	
40–52(M)/54(F)	25.7	24.3	
30–40	0	0.9	
< 30	0	0.9	
LV GLS, %	− 16.3 (2.7)	− 17.1 (2.4)	0.113
			0.580
≤ -16	66.7	71.7	
> -16	33.3	28.3	
RVEDD, mm	33.4 (5.5)	34.7 (4.6)	0.154
TAPSE, mm	20.6 (2.8)	21.1 (3.4)	0.382
			0.497
≤ 17	6	14	
> 17	29	97	
Sʹ, cm/s	13.3 (3.1)	13.5 (3.0)	0.640
			0.410
≥ 10	88.6	77.4	
< 10	11.4	16.2	
RV FAC, %	43.1 (9.4)	42.8 (8.5)	0.891
			0.260
≤ 35	25.7	17.1	
> 35	74.2	82.9	
RV strain, %	− 23.9 (4.0)	− 23.6 (5.2)	0.759
			0.419
≤ − 23	63.3	55.0	
> − 23	36.7	45.0	
*Diastolic dysfunction grade*, %			0.515
0	60.0	59.5	
I	40.0	36.9	
II	0	3.6	
III	0	0	
E/A	1.0 (0.6)	0.94 (0.4)	0.471
Eʹ average, cm/s	9.5 (3.3)	8.7(3.1)	0.202
E/Eʹ	6.4 (2.0)	8.0 (3.4)	0.013
LAVI, ml/m^2^	27.2 (10.4)	29.2 (10.3)	0.314
TR gradient			0.907
≤ 34 mmHg	95.2	94.6	
> 34 mmHg	4.7	5.4	

Data are presented as mean (SD), or as percentage

*ICU* intensive care unit, *LAVI* left atrial volume index, *LVEDV* left ventricular end-diastolic volume, *LVESV* left ventricular end-systolic volume, *LVEF* left ventricular ejection fraction, *LV GLS*, left ventricular global longitudinal strain; *RVEDD*, right ventricular end-diastolic diameter, *RV FAC* right ventricular fraction area change, *RV strain*, right ventricular strain, *TAPSE* tricuspid annular plane systolic excursion, *TR gradient* tricuspid regurgitation gradient

## Discussion

In the present study extensive echocardiographic evaluation at 6 weeks post-discharge in COVID-19 patients showed reduced LV and RV function in a notable amount of the patients. In 27% of patients an reduced LVEF was observed, accordingly 30% of patients LV GLS was reduced. Similarly RV function was impaired in a considerable number of patients as demonstrated by an abnormal TAPSE in 14% of patients, an reduced RV FAC in 18% of patients and abnormal RV strain in 43% of patients. However, LV or RV dysfunction was not related to residual symptoms at the outpatient clinic 6 weeks after discharge as assessed by dyspnoea on exertion with NYHA class. Furthermore, there were no echocardiographic differences between the first and second COVID-19 waves.

Previous studies have described differences in patients admitted during the first or second wave, and report that the second wave patients have less comorbidities, less severe disease, and possibly better medical management. [17, 25]. In our patient population at the LUMC similar observations were made. Patients admitted during the first wave had on average a more pronounced inflammatory response, represented by a higher maximum CRP, and more often intensive care unit admission. In addition, they had more often pulmonary embolism; reflecting a clinically more ill population. Several factors may play a role in this observed difference, e.g., better medical management, less severe infections and less frequent comorbidities in the second wave. This was reflected (although not significantly by a better functional status at 6 week (i.e., higher number of patients in NYHA class I) Despite these differences between patients admitted during the first and second wave, extensive echocardiographic evaluation of LV and RV function at 6 weeks post-discharge showed no significant differences. It would have been of particular interest to perform multi-variate modeling to evaluate which parameters are correlated to the occurrence of myocardial dysfunction. However, the number of patients included in the current registry do not allow for such advances analyses.

Several studies have reported echocardiographic abnormalities at follow-up after COVID-19 hospitalization. Subclinical LV dysfunction (measured with LV GLS) was observed in approximately one-third of patients at the one-month follow-up after hospitalization for COVID-19 and was also associated with elevated troponin T levels [13]. However, the cut-off applied for GLS (> − 18%) is a relatively low threshold thus incorporating patients with only mildly impaired LV strain. Akkaya et al. reported in non-hospitalized COVID-19 patients in the second wave at 3 months follow-up decreased RV-GLS and RV free wall longitudinal strain [14]. However, in their study the observed differences were small and of questionable clinical significance. Especially, no comparison was made according to common cut-off values. Ozer et al. reported subclinical RV dysfunction at 3 months follow-up of hospitalized COVID-19 patients in the first wave [15]. Similar to the previously mentioned study, the observed differences were small and no difference in LV function could be demonstrated. A clear relation between disease severity (concomitant pneumonia and need for steroid-therapy) was observed based on which the authors concluded that their findings may reflect the overall effects of severe disease, not specific to the virus. In our study, no differences in LV and RV function between first and second wave patients could be demonstrated, even though “first wave patients” were significantly more critically ill. Cardiac evaluation during hospitalization was not performed and potentially, cardiac dysfunction could be present during hospitalization and restored at time of out-patient follow-up. Van den Heuvel et al. reported a trend towards normalization in myocardial function 4 months post-discharge in patients during the first wave [18]. They reported no relation between troponin T levels during hospitalization and cardiac function at 4 months follow-up. Similar to the present study no relation between cardiac symptoms and myocardial dysfunction could be established. Similarly, no differences could be established between ICU patients or patients admitted to the general ward. There are many mechanisms by which COVD-19 might cause cardiac abnormalities: direct viral effects leading to myocyte damage, hypoxia induced damage, damage caused by increased afterload because of respiratory distress, pulmonary embolisms, the inflammatory response and damage caused by the auto-immune response [26–28]. In previous clinical studies severe cardiac injury and echocardiography abnormalities were observed. For instance Li et al. demonstrated a significant mortality in patients with impaired RV strain during admission for COVID-19 [29]. Similarly, Giustino et al. reported significant higher in-hospital mortality up to 35% in patients with both myocardial injury (troponin elevated above normal limit) and echocardiographic abnormalities [9]. It should be noted that in many of the above mentioned studies focussing on COVID-19 survivors, few patients had significant myocardial injury (i.e., troponin elevated more than three times the upper limit of normal). This could largely explain the limited incidence of significant myocardial dysfunction at out-patient follow-up. Furthermore, it deserves mentioning that in the present study echocardiography was only performed at the out-patient clinic, after admission. As a result information on myocardial function before or during admission is not available. This hampers further analyses on change over time.

In conclusion, 6 weeks post-discharge mild echocardiographic abnormalities both in LV and RV function were observed in a substantial amount of patients, however these were not correlated with NYHA class. In addition, echocardiographic parameters at follow-up do not differ between the first and second COVID-19 wave patients although the first wave patients were more ill reflected by several parameters. The absence of the correlation between echocardiographic parameters and NYHA class suggests that long term symptoms post-COVID might not be explained by the mildly abnormal cardiac function. The present analysis demonstrates that routine out-patient screening for cardiac abnormalities in all COVID-19 survivors is of limited clinical relevance. The majority of patient showed normal myocardial function at 6 weeks. However, potentially in a specific sub-group of patients with significant cardiac injury, e.g., patients with markedly elevated troponin T levels during admission, additional follow-up including echocardiography at the out-patient clinic could be relevant. We suggest to reserve cardiac screening only to patients with clinical suspicion of important cardiac dysfunction based on ECG and troponin levels.

### Supplementary Information

Below is the link to the electronic supplementary material.

Supplementary file1 (DOCX 24 kb)
