# Supplementary material for: Myocardial function in COVID-19 patients after hospital discharge: a descriptive study comparing the first and second ‘wave’ patients
Source: Int J Cardiovasc Imaging. 2022 Apr 16;38(9):1951–60. doi: 10.1007/s10554-022-02590-3 (PMC9013213; doi:10.1007/s10554-022-02590-3)
Supplement: Supplementary file 1 — Supplementary file1 (DOCX 24 kb) [file 10554_2022_2590_MOESM1_ESM.docx]

| **Supplementary Table 1. Baseline characteristics of the study population according to disease severity.** | | | | |
| --- | --- | --- | --- | --- |
|  | *All* | *ICU patients (n=35)* | *Non-ICU patients (n=111)* | P-value |
| Age, years | 61.1 (12.2) | 55.9 (10.7) | 62.7 (12.3) | **0.004** |
| Sex, men, % | 63.7 | 62.9 | 64.0 | 0.905 |
| BMI, kg/m^2^ | 28.6 (5.5) | 28.0 (5.1) | 28.8 (5.6) | 0.419 |
|  |  |  |  |  |
| *History of* |  |  |  |  |
| Hypertension, % | 34.9 | 25.7 | 37.8 | 0.190 |
| Diabetes, % | 23.3 | 28.6 | 21.6 | 0.396 |
|  |  |  |  |  |
| Cardiovascular disease, % |  |  |  |  |
| Atrial fibrillation/ atrial flutter | 6.8 | 2.9 | 8.1 | 0.284 |
| CVA/TIA | 4.8 | 5.7 | 4.5 | 0.770 |
| PVD | 2.7 | 2.7 | 2.9 | 0.961 |
|  |  |  |  |  |
| CKD, % | 8.2 | 14.3 | 6.3 | 0.134 |
| Smoking, % | 15.1 | 17.1 | 14.4 | 0.694 |
|  |  |  |  |  |
| *In hospital* |  |  |  |  |
| CRP maximum, mg/L | 145.3 (125.4) | 296(139) | 97 (71) | **<0.01** |
| Pulmonary embolism, % | 14.4 | 34.3 | 8.1 | **<0.01** |
| Troponin T max ng/L | 15.8 (16.3) | 21.4 (15.0) | 13.4 (16.3) | **0.020** |

Data are presented as mean (SD) or percentage.

BMI, body mass index; CKD, chronic kidney disease; CRP, C-reactive protein; CVA, cerebrovascular accident; ICU, intensive care unit; PVD, peripheral vascular disease; TIA, transient ischemic attack
